# Chaplains in Psychedelic Assisted Therapy: Rationale and Competencies

**DOI:** 10.1007/s10943-026-02591-9

**Published:** 2026-02-28

**Authors:** Stephen P. Lewis, Jaime Clark-Soles, Oriana Mayorga, Sarah K. Sawyer, Eliza Slavet, B. Jeffrey Vidt, Saundra Shanti, Rhiana Wiggins, Kathleen Willis, Jonathan Frey, R. Mark Grace, Aaron D. Cherniak

**Affiliations:** 1 University of California, San Diego, 200 West Arbor Drive, #8959, San Diego, CA 92103 USA; 2 Perkins School of Theology, Southern Methodist University, Dallas, USA; 3 Emory Center for Psychedelics and Spirituality, Emory University, Atlanta, USA; 4 Yale University, New Haven, USA; 5 Boulder, USA; 6 Vitas Healthcare, San Diego, USA; 7 Children’s Hospital of Eastern Ontario, Ottawa, Canada; 8 Salt Lake City, USA; 9 Harvard Divinity School, Harvard University, Cambridge, USA; 10 DAWA Entheogenic Education and Practices, Medford, USA; 11 Sharp HospiceCare, San Diego, USA; 12 Baylor Scott and White Health, Dallas, USA; 13 Stockholm University, Stockholm, Sweden; 14 Israel Center for Addiction and Mental Health, Hebrew University of Jerusalem, Jerusalem, Israel

**Keywords:** Psychedelic-assisted therapy, Psychedelics, Chaplain, Spiritual care provider, Spiritual health practitioner, Spiritual care, mental health, psychedelic chaplaincy, Psychedelic clinical trials

## Abstract

The rapid expansion of psychedelic research has opened significant pathways of opportunity in treating mental health disorders. Participants in recent clinical trials consistently report mystical-type experiences during dosing sessions, and some scholars posit that such experiences themselves help mediate clinical improvements in depression, PTSD, anxiety, and substance use disorders. These reports are in keeping with the historical reality that the use of psychedelic substances and plant medicine has been tied to spiritual experience and shamanic ceremonial practice. Professional clinical chaplains are trained and experienced in providing spiritual and emotional support for people encountering illness, life change, and trauma. They regularly assist participants who seek to make meaning of their experiences in the context of their own beliefs and spirituality. This article argues that as subject matter experts in spirituality and health, chaplains are an asset to psychedelic assisted therapies and should be utilized in research trials and clinical practice, especially given a relative lack of training in spirituality and religion among interprofessional practitioners. We offer this set of competencies for chaplains to provide high quality, safe, and ethical care in the context of psychedelic medicines. These competencies include spiritual and religious care, spiritual inquiry, empathic presence, ethical engagement, and advocacy. We recognize that chaplains will need to pursue specialized education and supervised experience beyond their standard professional requirements, and pathways of personal preparation are discussed. The presence of qualified chaplains will help ensure that participants navigate non-ordinary states of consciousness with safety, sensitivity, and insight regarding improvement and growth.

## Introduction

Professional clinical chaplains provide spiritual and emotional support to patients across the lifespan and in all areas of medical specialty, including mental health (Timmins et al., 2018). Decades of evidence continue to document the activities, perceptions, utilization, and efficacy of chaplains in spaces that include hospitals, hospice, the military, corporations, and more (Fitchett, 2017; Handzo et al., 2014). Chaplains offer spiritual practices (religious and non-religious) such as prayer and guided meditation, connections with community clergy, rituals to create meaning and a sense of the sacred, conversations with patients that clarify values and how they align with healthcare decision-making, conversations around “practical matters” such as finances or family dynamics, and compassionate presence during emotionally difficult situations (Fitchett, 2017; Idler et al., 2015). With recent increases in psychedelic research and expanding access to decriminalized treatments, there is a growing interest by researchers and clinicians in chaplains providing support in this context as well (Beachy & Petersen, 2022; Peacock et al., 2024). In this article, we argue that the presence of professional chaplains in psychedelic assisted therapy (PAT) is invaluable to the field and to those under the care of this emerging treatment modality. We also recognize that PAT is a novel application of professional clinical chaplaincy, which requires the development and practice of specific competencies in this context.

Archaeological evidence from many cultures around the world suggests that psychoactive plants and fungi have been utilized for thousands of years in burial rituals, shamanic healing, and other religious contexts (Samorini, 2019), all familiar territory for modern day chaplains. The modern, Western explorations of the ‘psychedelic experience of mystical consciousness’ (Pahnke & Richards, 1966, *p*. 176) began in the past hundred years, with the synthesis of substances such as lysergic acid diethylamide (LSD) and 3,4-Methylenedioxy methamphetamine (MDMA). Even in the setting of patently scientific investigation, themes that chaplains are well-familiar with emerged (Beachy & Petersen, 2022).

This period of research concluded in the 1970s when restrictive governmental policies effectively ended meaningful research for the next 20–30 years (Doblin et al., 2019). Recent years, however, have seen a resurgence of research on psychedelic medicines and their potential to treat a wide variety of mental health conditions. Studies involving the use of MDMA, psilocybin, LSD, ketamine, and N,N-Dimethyltryptamine (DMT) have produced significant clinical improvements in individuals with PTSD (Mitchell et al., 2021, 2023), major depressive disorder (Metaxa & Clarke, 2024), anxiety disorders (Gasser et al., 2014; Holze et al., 2022), and substance use disorders (Bogenschutz et al., 2022; Garcia-Romeu et al., 2019), among others. In response to the growing body of research, the US Food and Drug Administration has granted breakthrough therapy designation to MDMA, psilocybin, and LSD (though at the time of this writing, none have been licensed). While the potentially positive effects are hopeful for some, it is worth noting that some PAT participants have reported negative effects, including suicidality (Goodwin et al., 2022). Nevertheless, these studies suggest that PAT represents a compelling approach to mental health treatment and as we argue below, mystical/spiritual factors may play a significant role. Through the study of psychedelics, scientific research may be engaging spirituality in previously unexpected ways.

## Psychedelics, Mystical/Spiritual Experience, and the Role of Chaplains

Numerous studies suggest that mystical experiences may play an important role in the clinical improvements made by participants in PAT. In calling attention to the need to thoughtfully understand the implications of spiritual experience in healthcare, Puchalski et al. (2009) define spirituality as “the aspect of humanity that refers to the way individuals seek and express meaning and purpose and the way they experience their connectedness to the moment, to self, to others, to nature, and to the significant or sacred.” With psychedelics, “profoundly sacred experiences are now occurring in the laboratories of medical professionals and social scientists” (Richards, 2015, *p*. 13). These compounds have been found to reliably produce “mystical-type experiences” (including aspects of unity, sacredness, noetic quality, positive mood, ineffability, paradoxicality, and transcendence of time and space, as described by Stace (1960)) and mediate observed clinical improvements (Griffiths et al., 2006, 2008). A 2006 study with religiously/spiritually active adults at Johns Hopkins University found that “when administered to volunteers under supportive conditions, psilocybin occasioned experiences similar to spontaneously occurring mystical experiences and which were evaluated by volunteers as having substantial and sustained personal meaning and spiritual significance” (Griffiths et al., 2006). Two-thirds of the study participants rated their psilocybin experience as either the single most meaningful experience of their lives, or in the top five most meaningful experiences, on level with the birth of a child or the death of a parent. One-third of the participants rated the experience as the most significant spiritual experience of their life (Griffiths et al., 2006).

Another example of experiences that some might characterize as ‘spiritual’ or ‘religious’ can be found in survey data published in 2020, which reports “entity encounter experiences” among respondents who had inhaled DMT (Davis et al., 2020). The respondents described interactions with “entities” using terms such as “being,” “spirit,” and “guide” and more than half of the respondents counted these experiences among the most meaningful of their lives. This, and another survey study report significant declines in respondent self-identification as atheist following experiences with psilocybin, LSD, ayahuasca, and DMT (Davis et al., 2020; Griffiths et al., 2019).

Multiple published review articles draw correlations between the mystical experience and improved outcome measures for issues including depression, anxiety, alcohol use disorder, and smoking cessation among others (Garcia-Romeu et al., 2019; Holze et al., 2022; Johnson et al., 2017; Ross et al., 2016). Also, mystical experience scores were predictive of treatment success at long-term follow-up (Yaden & Griffiths, 2021). One review found that “significant correlation between mystical experience and clinical improvement was established in nine out of twelve studies analyzed for short- and medium-term results” (Ko et al., 2022). Two more recent reviews challenge the assumption of correlation between mystical experience and clinical improvement, instead crediting participants’ psychological insights and emotional breakthroughs gained as the primary drivers of positive change. In some reviewed studies, therapeutic effects of psychedelics were more strongly associated with “acute insight” than with mystical-type experiences, whereas others found that mystical-type experiences were more strongly associated with therapeutic outcomes than insight, and some showed mixed results (Kangaslampi, 2023; Kugel et al., 2025). These reviews help make an important point: clinical improvements do not require mystical experiences per se. In fact, the ways in which the terms “mystical” and “spiritual” are defined and characterized within PAT has been questioned related to either a lack of clarity or to underlying religious perennialism (Baker et al., 2023; Mosurinjohn et al., 2023). As the re-emergence of PAT studies is still nascent, it is possible that less loaded conceptualizations such as “connectedness” (Watts et al., 2022) may come to take on greater importance, but that is beyond the scope of this article.

Palitsky, et al. (2023) argue that because of the prevalence of spiritual, existential, religious, and theological (SERT) experiences of participants in psychedelic studies, more attention needs to be paid to integrating these elements in PAT. The authors argue, “SERT experiences are important not only because they are common in PAT, but also because of their potential role as treatment mediators. Careful attention to mediators of therapeutic change is crucial for the development and optimization of effective behavioral treatments” (*p*. 744). Further, if SERT dynamics continue to act as mediators in PAT, they should be attended to in a systematized, pluralistic, and scalable way.

Most commonly in PAT, psychological support is provided by licensed psychologists, social workers, and therapists (Beachy & Petersen, 2022). This support usually consists of treatment team members present with participants in one or more “preparation” sessions prior to dosing sessions, during dosing sessions, and in “integration” sessions following dosing sessions. Preparation sessions may include rapport-building between participants and team members, education, and articulation of participants’ intentions. Integration sessions consist of meaning-making conversations regarding participant experiences of dosing sessions and discussions of implications of insights (Aday et al., 2024). Palitsky et al. (2023) cite a lack of consistency in the definition and application of psychotherapeutic components in psychedelic studies to date. Even if there were more consistent therapeutic protocols in place, Palitsky et al. (2023) go on to note that among clinical therapists, relatively few have received any kind of formal education or instruction in working with issues of religion and spirituality. Vieten and Lukoff (2022) cite multiple studies of psychology training programs and students, finding that 75% of training programs offer no courses in religion and spirituality, and that these issues are among the most neglected topics of study.

Palitsky et al. (2023) lay out a framework for addressing issues in the treatment process, including attending to participants’ religious and spiritual beliefs, cultural backgrounds, social relationships, and developmental history. While noting the importance of competent therapists in applying psychotherapeutic principles, they argue for the inclusion of qualified “spiritual health practitioners” (chaplains) on treatment teams. Board certified chaplains bring significant professional preparation to their work – including a minimum three-year post-graduate experience, at least 1600 hours of Clinical Pastoral Education (CPE), the submission of position papers on 29 professional competencies, and board interviews. To be clear, the fact that people undergoing psychedelic care are consistently encountering spiritual/mystical experiences calls for the presence of chaplains as trained and skilled clinicians, *regardless* of whether these mystical experiences are mediators of clinical improvements. Failure to support the SERT dynamics unnecessarily exposes participants to adverse events, such as struggles related to religious worldview or cultural background (Palitsky et al., 2024). We would also note that inasmuch as PAT is taking place in clinical settings where chaplains already participate as interprofessional team members, it is appropriate for them to be involved, even when PAT participants have not encountered experiences they might describe as mystical or spiritual. The emotional support, meaning-making, and potential support for adverse events offered by chaplains holds value.

A qualitative analysis of the work of spiritual health practitioners (chaplains) in PAT highlights their unique contributions, including competency to work with spiritual material, awareness of power dynamics, familiarity with non-ordinary states of consciousness, holding space with clients, and the ability to offer a counter-balance to the biomedical perspective by opening space to discuss ideas that may come into conflict with the underlying assumptions of medical research and clinical practice (Peacock et al., 2024). There are studies underway that utilize chaplains as members of interdisciplinary teams, including phase three clinical trials investigating MDMA-assisted therapy for the treatment of PTSD (Mitchell et al., 2023), and palliative care psilocybin studies at the Dana-Farber Cancer Institute (Beaussant, 2023) and Emory University (Zarrabi, 2023). A critical evaluation of treatment protocols in one of these studies argues that the presence of chaplains is appreciated by patients and adds to feelings of confidence in the treatment team (Palitsky et al., 2025a, 2025b).

## Method

The competencies presented below represent the efforts of a working group of professional chaplains, researchers, and educators affiliated with a research network under the umbrella of the Transforming Chaplaincy research think tank known as the Psychedelic Care Network. This working group met over a two-year period to review current competency models in the fields of chaplaincy and PAT to define both shared and distinct practices of chaplains working alongside other providers in the context of psychedelic medicine. The existing competency models considered include the 29 competency areas of the Common Qualifications and Competencies of the Board of Chaplaincy Certification, Inc. (Common Qualifications and Competencies, 2016); the 18 outcomes and 88 indicators of the Outcomes and Indicators for Clinical Pastoral Education of the Association for Clinical Pastoral Education (CPE) (Objectives and Outcomes for Level I/Level II CPE—ACPE Manuals—2020, 2020); Janis Phelps’ “Developing Guidelines and Competencies for the Training of Psychedelic Therapists” (Phelps, 2017); and Rochester, et.al.’s “Entheogens and Psychedelics in Canada: Proposal for a New Paradigm” (Rochester et al., 2022).

The working group organized the work of those who developed the models above on a crosswalk comparison table to identify areas of uniqueness and similarity among the elements of competency. Rather than duplicating the already well-established skills and behaviors defined elsewhere, the working group defined what makes the competencies below distinct to the practice of chaplaincy in the context of psychedelic medicines. In areas of noticeable overlap between therapist competencies and the chaplaincy competencies identified here, the working group defined how the latter are practiced in unique ways by professional chaplains. Similarly, competencies that are already common to the practice of professional chaplaincy may look different when practiced in the context of PAT. Finally, the group identified important areas of competency that did not exist in other models.

In examining the competency models, the group consolidated these competencies under thematic categories and skills of practice. We agreed that thorough but concise competency statements would make the most useful tools and allow for smoother application in practice. Common chaplaincy competencies that have not been reiterated here include broad areas such as integration of theological, psychological, and academic theory in practice; professional identity and conduct; professional practice skills (triage, support for grief process, public services and rituals, documentation); and organizational leadership (Common Qualifications and Competencies, 2016). The group recognizes that these are not granular descriptions of techniques or behaviors, but rather explanations of the kinds of learning and skills that chaplains working in this specialty area should master. Importantly, these competencies are also best understood as extending beyond other forms of preparation common to chaplaincy (i.e. education, CPE).

**Fig. 1:**
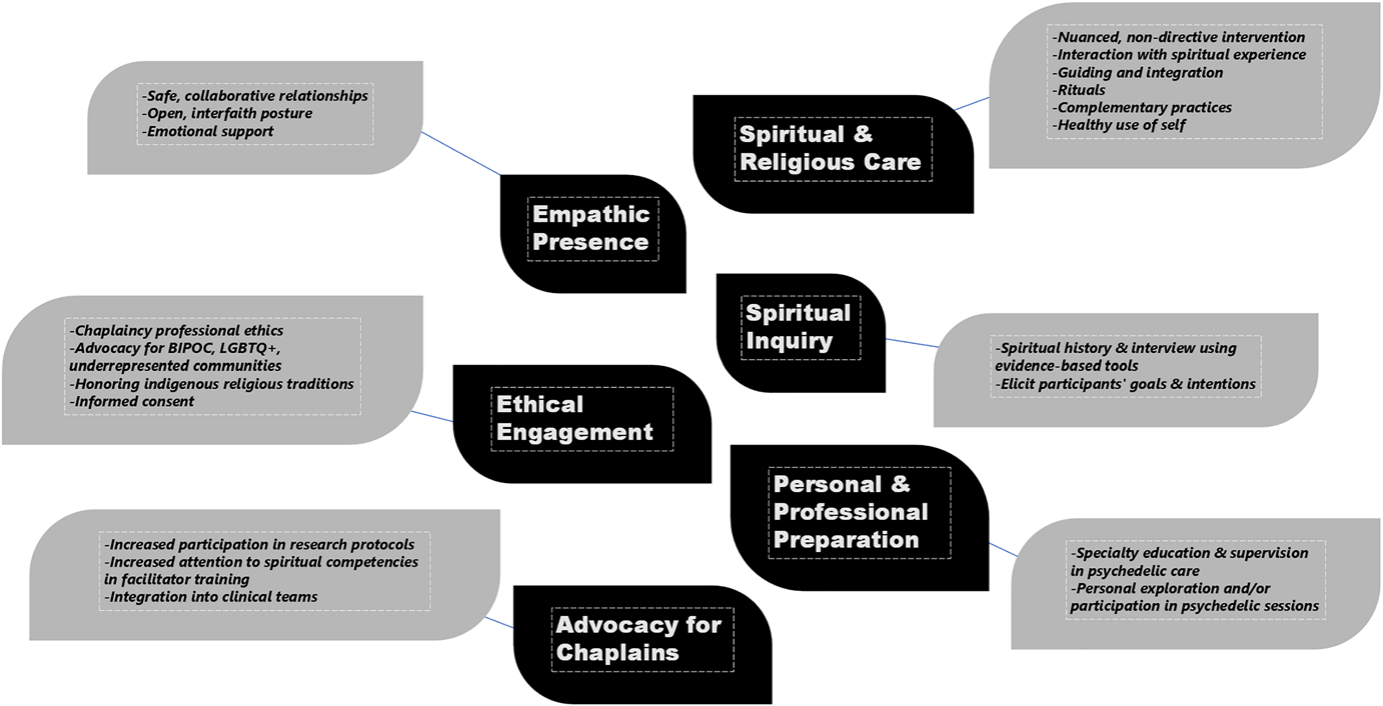
Chaplain competencies in psychedelic-assisted therapy

The working group made several presentations of an initial set of competencies in conference and webinar settings throughout 2023, where feedback and critique were sought, received, and recorded for further discussion. Revisions were made to emphasize the non-linear, non-hierarchical nature of the competencies, and to include previously unaddressed dimensions of care. The working group offers the competencies below with gratitude for those who developed foundational work referenced here, and with the recognition that as psychedelics become more widely researched and utilized in clinical environments, further additions and adjustments may become necessary in time. See Fig. 1 for a visual representation.

Before beginning, we would like to acknowledge the use of particular terms. We chose to use the term “chaplain” here with the understanding that others have used “spiritual health practitioner,” “spiritual counselor,” or “spiritual care provider,” as chaplain is the term of clinical specialty as defined by the certifying boards of our profession. We also recognize the potential for confusion in our use of “therapy” here, as in psychedelic-assisted therapy. We include it as the term most commonly used in psychedelic research and clinical practice environments where supportive care, but not necessarily psychotherapy, is involved. Relatedly, terms such as “therapist,” “guide,” “monitor,” or “coach” are also widely used in PAT literature to refer to those who provide support for participants (Palitsky et al., 2025a). We will use the term “facilitator(s)” when referring to non-licensed clinical support providers to avoid confusion and out of respect for our interprofessional colleagues, and in keeping with scope of practice.

This article is an expression of a generalist-specialist model used in other disciplines. Miller et al. (2023) note that in PAT, as in hospice and palliative care, all members of treatment teams function as care generalists, attending to education, descriptions of treatment effects, and adverse events, and when specialists are needed to address issues such as spiritual distress or meaning-making around religious beliefs, chaplains should be consulted, just as therapists should be consulted when issues like complex family dynamics or trauma-informed care emerge. We believe several of the competencies below apply to our spiritual care generalist colleagues. For example, generalists offer empathic presence, take spiritual histories, incorporate participants’ spiritual beliefs into their supportive care, show respect for indigenous practices related to psychedelics, and advocate for the presence of chaplains (Vieten et al., 2013). However, we developed these competencies specifically for in-depth use by chaplains as spiritual care specialists.

The competencies described here are to be understood as applying specifically to research and clinical environments where chaplains commonly work. We strongly suggest that chaplains working outside of clinical settings exercise caution and attention to ethical practices, harm reduction, and avoidance of religiously manipulative or coercive group dynamics.

## Chaplain Competencies

### Spiritual and Religious Care

#### Nuanced, non-directive intervention

Participants in the context of PAT are either experiencing a non-ordinary state of consciousness, preparing to do so, or attempting to understand their experiences in post-dosing integration sessions. In dosing sessions, they cannot expect to have a typical, rational, and/or linear conversation of questions and responses. Though a range of psychological interventions are being investigated and implemented in PAT research, most protocols encourage facilitators to use a non-directive approach, minimizing interventions during dosing sessions (Seybert et al., 2025). The participant is empowered to lead the process, rather than the facilitator imposing a pre-loaded agenda (Johnson, 2021). In CPE, chaplains develop expertise in holding space with compassion, patience, and steady strength, restraining impulses toward advice-giving, directive leadership, or imposing theoretical/theological constructs or mandates (Kidd, 2012). Chaplains encourage people to use the spiritual traditions and practices they already have and assist participants in exploring how they can transform and heal on their own terms. This process not only requires nuance and gentleness to understand underlying experiences and motivations, but also reassuring persistence to stay with participants through what may be very challenging experiences. Psychedelic practitioners from other disciplines often find this non-directive approach counter-intuitive and must adapt to and develop the skill of non-directive presence and allowing the participants to lead, especially when strong emotions are expressed (Phelps, 2017).

#### Ability to Interact with Spiritual/Mystical Experiences, Religious Ambivalence Regarding the Use of Psychedelics, and Religious Trauma

The ability to interact with spiritual and/or mystical material is central to chaplaincy, especially in the context of PAT (Peacock et al., 2024). Chaplains must maintain presence in a reassuring manner that communicates acceptance and safety. Chaplains may also help some participants work through their ambivalence regarding the use of psychedelics—for example, some are affiliated with religious traditions that prohibit the use of mind-altering substances, and they may experience a sense of guilt or shame related to the use of substances considered taboo (Exline et al., 2024). In some cases, participants may have perceptions that psychedelics provide a doorway to spiritual or supernatural messages (both positive and negative) (Exline et al., 2023). Professional training for chaplains would discourage telling participants that their received teachings are wrong, and in some cases where this ambivalence persists or presents as a risk for adverse effects, chaplains are advised to explore either delaying the participants’ use of psychedelics until this can be resolved or recommending non-psychedelic forms of therapeutic support. However, if they have made the decision to proceed despite their received traditions by engaging these therapies, there may be a need to help them contextualize and make meaning around this ambivalence.

Chaplains can be of particular help with those who have experienced trauma in religious settings. It might be quite disconcerting for them to process these traumatic events while having a powerful spiritual or mystical experience, especially if their trauma involved spiritual manipulation. Chaplains bring specific insight into how religious abusers exploit power dynamics and spiritual vulnerabilities. Participants in this context may require a renegotiation of how they experience spirituality as safe, healthy, and generative.

#### Guiding and Integration Practices

Chaplains, along with other facilitator colleagues, interact with participants throughout a process that begins well before dosing sessions and continues well after. In dosing sessions, facilitators are present to interact with participants, at times quietly observing, giving reassurance of safety; at times, encouraging participants to attend to inner experiences; at times, inviting participants to verbalize their feelings and thoughts (Mithoefer, 2015). Chaplains, especially in palliative care and hospice contexts, may be experienced in sitting with patients in non-ordinary states of consciousness—whether because of pain medications, dementia processes, or end of life phases. For example, patients may see relatives (living or dead), religious figures (benevolent or malevolent), and even have conversations with them (Hession et al., 2023). These experiences may be very frightening or reassuring. Chaplains should be comfortable working with these experiences rather than trying to distinguish the “reality” or “verifiability,” especially in the context of psychedelic medicines (Balboni et al., 2017). While facilitators from other disciplines may have an impulse to correct or re-ground participants in “reality,” chaplains are able help make meaning of these encounters. In integration sessions after dosing sessions, chaplains remain open as participants talk through and begin to make sense of what they encountered, especially if their experiences were challenging (Bathje et al., 2022; Gashi et al., 2021). Chaplains can also explore areas in which participants experience spiritual struggle through interacting with psychedelics—whether encountering or resolving struggle (Schutt et al., 2025). As noted above, the granular techniques and skills of guiding and integration are outside the scope of this article; however, the competency below, “Personal and Professional Preparation,” calls for chaplains to gain specialized training and supervised practice in this area.

#### Rituals

Chaplains are experts in the use of ritual (Klitzman et al., 2022), which can include attention to the physical, aesthetic space. Psychedelic research trials and clinical practice sessions typically take place in environments that have been made to feel as comfortable as possible, with sofas, artwork, plants, and candles. Participants may be invited to bring in certain items of personal meaning as sources of comfort—things like family photos, blankets, and objects of religious and/or cultural importance. Chaplains can help participants use or develop meaningful rituals based on their spiritual traditions. In the dosing session, the chaplain may offer a prayer, reflection, or blessing appropriate to the participant’s own spirituality. Chaplains will be sensitive to the importance of specific symbols. In preparation and integration sessions, chaplains can use prayer or guided meditation to invite the participants to interact with themselves, the facilitator team, and/or their sense of the divine in guiding them toward healing and growth.

In the context of clinical trials, valid concerns have been raised about inappropriate religious ideas being suggested to research subjects through both subtle and overt aspects of PAT treatment environments, such as an image of a Buddha or prayers (Johnson, 2021). This risks the potential for priming subjects with expectations or manipulating their own experiences. This competency calls on the expertise of chaplains in honoring the spirituality of the participant, rather than imposing outside religious or spiritual beliefs and practices upon them.

#### Familiarity with Complementary Practices

While it is important that chaplains maintain their scope of practice, they should also have familiarity with some of the psychotherapeutic models and skills commonly utilized in this treatment modality (Yaden et al., 2022). Some of the models and skills at play include the concept of the inner healer, meditation practices, trauma-informed care, somatic therapy, breathwork, and expressive arts (Franco Corso et al., 2023; Millière et al., 2018). It is notable that as PAT studies continue to develop and emerge, some theoretical frameworks (for example Internal Family Systems) are employed without a thorough evidence base, which warrants caution on the part of practitioners of all disciplines.

#### Self-awareness and Healthy Use of Self

Among the basic aims of CPE is “to develop students’ awareness of themselves as ministers and of the ways their ministry affects persons, and to develop students’ awareness of how their attitudes, values, assumptions, strengths, and weaknesses affect their pastoral care (Objectives and Outcomes for Level I/Level II CPE—ACPE Manuals—2020, 2020).” This kind of self-awareness is one area of distinction for chaplains relative to other therapeutic disciplines that engage in psychedelic care. While monitoring the dynamics of therapeutic relationships is a common skill for therapists, chaplains are trained to be mindful of a larger scope of influences, including their own spirituality, religious theologies, and family of origin practices. Chaplains should also maintain an awareness of their own motivations for work with psychedelics, and how that might impact the care they provide (Pasricha et al., 2025).

Self-care practices are also notable here. Unlike other contexts of care for therapists and chaplains, psychedelic dosing sessions regularly last six to eight hours, or even longer, depending on the substance. This presents substantial challenges in terms of maintaining presence and active engagement. All interprofessional facilitators must be skillful at checking in with themselves to respond appropriately, and whenever applicable, rely on the support of co-facilitators.

### Empathic Presence

Chaplains are skilled at establishing open, safe, compassionate, and trusting relationships. Chaplains describe their role and assure the participants that they will be supported on their own religious and spiritual terms. Non-religious or atheist participants may express discomfort with the presence of a chaplain, especially if they have encountered trauma in religious or spiritual contexts and may need assurance regarding the chaplain’s non-imposition of religious categories. If a participant remains uncomfortable with the presence of a chaplain, it may be appropriate to refer the participant to a non-chaplain facilitator, unless chaplains are part of a prescribed treatment protocol in a research study. In preparation sessions, chaplains explain that while not universally the case, these medicines have been known to result in experiences that some participants describe or interpret as “mystical” or “spiritual,” including by people who self-identify as atheist, agnostic, and committed materialists (Marks et al., 2024). Chaplains describe how participants can integrate these experiences without imposing theological frameworks incompatible with the participants’ own beliefs. Chaplains may also reiterate that any discussion of SERT experiences is voluntary in keeping with the non-directive posture of care.

The early conversations during screening, inquiry, and education sessions are key times for developing collaborative relationships. As participants describe their spiritual, mental health, social, and trauma histories, chaplains compassionately affirm their sources of comfort and support, and guide them in asking questions for exploration during their dosing sessions. Maintaining that sense of safety is critical during dosing sessions as participants may experience intense emotions, including joy, grief, anger, shame, and fear. In PAT, participants will often experience non-ordinary states of consciousness that are non-linear and/or non-rational. As chaplains attend to the participant’s experience during the dosing-sessions and integration, they model self-compassion and establish an open and safe space for healing.

### Spiritual Inquiry

Each discipline within the healthcare model has an approach to conducting assessments. Healthcare providers are familiar with the physician’s report on a patient’s medical history, the social worker’s interview assessing psycho-social and socioeconomic factors at play, and the chaplain’s exploration of a person’s religious backgrounds and/or spiritual beliefs and needs (Borneman et al., 2010; Fitchett & Risk, 2009; Shields et al., 2015). In the context of psychedelics, we intentionally chose the term “inquiry” rather than “assessment.” Briefly, this is to reaffirm the participant-led process of care. Chaplains may employ evidence-based spiritual screening and assessment tools, such as the Faith, Importance and Influence, Community, and Address (FICA) Spiritual History Tool (Borneman et al., 2010) or the PC-6 Model for Spiritual Assessment in Palliative Care (Labuschagne et al., 2025), but the language of inquiry communicates the value of accompaniment. Frequently in medical contexts, there is an assumed connection between assessment and outcome-focused treatment planning, which can include outcome-focused Chaplaincy interventions (Fitchett et al., 2014; Sprik et al., 2024). However, in the specific context of PAT, we believe it is important to de-link assessment with presumed outcomes. While there is an “agenda” of helping the participant experience reduced suffering, allowing the participant’s own agenda and experiences to guide the way as they engage their non-ordinary states of consciousness is central.

In preparation for PAT dosing sessions, chaplains conduct a spiritual and religious history, including attention to the participant’s spiritual background, beliefs, core emotional motivations, perceptions and needs. It is also crucial to learn if a participant remembers a previous challenging psychedelic experience or if their perceptions of psychedelics are shaped by religious prohibitions (Exline et al., 2023, 2024; Palitsky et al., 2024). Articulating how these elements shape their experience of medical and/or mental health conditions may elicit their hopes, fears, and spiritual assets for engaging PAT, and will give chaplains the ability to support participants in their own goals.

Frequently, the evaluation of mystical-type experiences that research study subjects encounter is produced retrospectively through instruments like the Mystical Experience Questionnaire, and according to the latent definitions of “mystical” (Barrett et al., 2015; Pahnke, 1969). In research protocols, relatively little emphasis has been given to inquiry of spiritual experiences prior to the dosing sessions. When questionnaires are administered prior to dosing sessions, they are given alongside other psychiatric, physical, and psychological instruments with little to no follow-up outside of study exclusion criteria. Continuing to neglect these aspects of histories poses potential safety risks. For example, participants’ prior mystical or spiritual encounters might condition the way they experience non-ordinary states in dosing sessions and make them vulnerable to adverse events (Cherniak et al., 2023; Palitsky et al., 2024). Future research and treatment protocols should include the development of new instruments to gather history and attitudes toward spirituality, mystical experiences, and theological constructs, as well as how these shape participants’ experiences of spirituality both during and after the dosing sessions.

### Ethical Engagement

#### Abiding by Established Ethical Standards

Chaplains are responsible for ensuring that they are familiar with and vigilant adherents to high ethical standards (Council on Collaboration, 2004). Religious leaders and chaplains have been heavily involved in the development and implementation of medical ethics over the past sixty years, so it is clear that there are responsibilities in this specific expression of care as well (Beauchamp & Childress, 2019). Unfortunately, the history of psychedelics in popular culture and in medical research has many examples of poor ethical practice and outright abuse (Grof et al., 2017), as well as sexual abuse and boundary violations during PAT clinical trials (McNamee et al., 2023). Ethical practice and reporting of ethical violations are the responsibilities of all chaplains in all contexts of care, but especially in PAT.

A common code of ethics for spiritual care providers has been agreed upon by the major certifying bodies of chaplains, including the Association of Professional Chaplains, Neshama: Association of Jewish Chaplains, National Association of Catholic Chaplains, Canadian Association for Spiritual Care, and the Association for Clinical Pastoral Education (Council on Collaboration, 2004). Several of these standards are particularly pertinent to the practice of PAT, such as respect for cultural and religious values of participants, mindfulness of the imbalance of power, avoidance of conflicts of interest, avoidance of sexual misconduct, and acting with attention to scope of practice. People interacting with non-ordinary states of consciousness are particularly vulnerable and suggestible, so issues of power imbalance must be closely guarded.

As popular media sources have increased cultural awareness of psychedelics, their potential for addressing mental health issues, and their spiritual uses, increasing numbers of individuals are expressing interest in participating. We assert that a harm reduction approach would be helpful for chaplains faced with participant inquiries about the unsanctioned and unsafe use of psychedelics. This allows for a non-judgmental posture, respect for autonomy, and education (Pilecki et al., 2021).

Additionally, chaplains have a role to play in the way ethics are practiced by interdisciplinary colleagues. For example, chaplains are frequently cautioned to maintain scope of practice in terms of not attempting to practice psychotherapy with patients. We argue that scope of practice goes both ways, and the frequency of mystical/spiritual experiences participants have with psychedelic medicines puts therapists who are untrained in spirituality and religion in a position of potentially working outside of their expertise (Vieten & Lukoff, 2022). Chaplains can gently, but firmly remind interdisciplinary colleagues to allow the subject matter experts to do the work they have been trained to do.

#### Advocacy for BIPOC, LGBTQ + , Marginalized, and Underrepresented Communities

Despite the expansion of PAT research and calls for greater inclusion of ethnoracial minoritized populations, a 2024 systematic review of clinical trials indicates that White, non-Hispanic participants remain an over-represented population (Hughes & Garcia-Romeu, 2024). One of the significant challenges in the medical model of PAT research and practice is that it is very expensive due to the extensive therapeutic support models involving multiple lengthy sessions (Williams & Labate, 2019). Chaplains are uniquely positioned to advocate for people who may lack access to funding or the social capital necessary to receive this care, just as they have done in other aspects of healthcare disparities (Sharma & Reimer-Kirkham, 2023). This includes advocacy for religiously underrepresented groups (Rab et al., 2025). Ironically, it is members of these marginalized communities who historically have higher levels of exposure to the mental health conditions that psychedelic assisted therapies are attempting to address (Smith et al., 2022). Cultural humility and cultural competency will help chaplains respectfully respond to inequities and develop trusting relationships.

#### Honoring of Indigenous Religious and Cultural Traditions

Although psychedelics have only come to the attention of Western countries in the past hundred years, these substances have been used in shamanic, ceremonial, and religious contexts for several thousand years (Urrutia et al., 2023). Chaplains are responsible to honor both the beliefs and practices of the patients they serve, and the spiritual traditions of the original practitioners. Competencies for board certified chaplains require awareness of a wide variety of faith traditions, and in the context of plant medicines, it is important to honor these practices. There is an unfortunate history within the field of psychedelics of religious appropriation, commodification, and misuse of the spiritual traditions (Celidwen et al., 2023). There is also an ecological risk of depleting the natural resources from which certain substances are obtained (Uthaug et al., 2019). Chaplains have a role to play in speaking out on these issues, especially in calling for respect for indigenous practices in clinical environments, modeling resistance to colonial practices, honoring cultural and religious ways, and practicing “two-eyed seeing” – a way of incorporating indigenous and Western sources of knowledge to the benefit of the community (Iwama et al., 2009).

#### Informed Consent

Chaplains are responsible for ensuring that participants are fully educated about the benefits and risks associated with the medicines they are taking. When working with co-facilitators, this is a shared responsibility that is often attended to in screening or preparation sessions prior to dosing. Informed consent is not a common topic of skill or expertise for chaplains, so this is one area where the competency of “Personal and Professional Preparation” becomes important. Marks et al. (2024) have offered a thorough list of considerations that psychedelic practitioners should heed with respect to informed consent, including perceptual changes, vulnerable states, and personality changes.

All caregivers should know the common physical effects and risk profiles of psychedelic substances. Chaplains do not speak to the neurobiological effects of the medicines (and should offer referrals to qualified specialists to answer questions), but they can speak to the common mystical/spiritual dynamics in non-ordinary states of consciousness. They need to be clear that psychedelic medicines may elicit difficult, challenging experiences. Even when challenging, the experiences participants encounter may still be deeply meaningful and part of the growth experiences they describe (Barrett et al., 2016; Griffiths et al., 2006). When participants express concerns about this, it is imperative that chaplains affirm the participants’ autonomy and not coerce them to do things that make them uncomfortable. If a chaplain has concerns about the participant’s suitability for PAT, this needs to be discussed transparently with the individual (Palitsky et al., 2024).

Chaplains can also normalize the fact that non-ordinary states of consciousness may (or may not) alter the spiritual beliefs of participants, or at least the way those beliefs are understood (Griffiths et al., 2019; Nayak et al., 2022). Unexpected shifts in belief may be disorienting and potentially spiritually de-stabilizing. It is also notable that some participants may desire to encounter mystical/spiritual experiences with PAT and be disappointed if this either does not occur or is less impactful than hoped for. Belief change may also take place separate from non-ordinary states of consciousness, whether as a result of therapeutic support from PAT facilitators or the neurobiological effects of the psychedelic substances. Whether or not belief change or spiritual experience is present, as spiritual care specialists, the role of chaplains here is of great value in preparation, dosing, and integration sessions (Cherniak & Granqvist, 2025).

Another important aspect of informed consent in the context of PAT is the use of non-sexual physical touch. Especially during dosing sessions, if a participant becomes frightened or uncomfortable, it can be reassuring for them to hold the hand of a facilitator or have a gentle hand placed on their shoulder. Physical touch must be addressed before dosing sessions, and issues of consent must be as clear as possible (Marks et al., 2024). In a “normal” therapeutic environment, touch that is not connected with a physical assessment or intervention is uncommon, so chaplains need to proactively and transparently discuss this issue to assess participants’ wishes. If a participant says in a preparation session that they do not want to be touched, that should be honored. In this case, though, the participant should be made aware that while under the influence of the medicines, people sometimes change their mind about touch—how this should be handled needs to be agreed upon ahead of time. This is an important part of building a trusting relationship with the participant. While there are no formal or specific professional directives regarding physical touch, healthcare chaplains may be accustomed to holding hands or hugging care seekers experiencing fear or grief. In the context of PAT, though, informed consent is needed to articulate care for the suggestibility and vulnerabilities of PAT participants. Adhering to the Common Code of Ethics for Chaplains, Pastoral Counselors, Pastoral Educators and Students, which calls for attention to power imbalances and refraining from sexual behavior between chaplains and participants, is critically important (Council on Collaboration, 2004).

#### Navigating Changing Legal Landscapes

Chaplains who wish to devote attention to care in the current context of psychedelics are faced with unique challenges. At the time of this writing, there are relatively few opportunities for PAT practice in legal settings, with the exception of ketamine clinics and clinical research trials. While the authors do not endorse these competencies for use in unsanctioned settings, we do recognize that some chaplains may choose to practice there. In some ways, the ethical challenges are similar to those previously confronted by chaplains in issues such as euthanasia, abortion, gender affirming care, and the complex landscape of mandatory reporting laws (Farr et al., 2023; Wirpsa et al., 2024). This involves reflection on personal values, religious and cultural teachings, and justice concerns. Chaplains are responsible to be familiar with pertinent laws and regulations and to exercise firm boundaries around discussions of sourcing substances or encouraging illegal use.

### Personal and Professional Preparation

To provide competent care, chaplains and other interdisciplinary team members will need to engage in specific education and training (Phelps & Henry, 2022). Chaplains need to have a general understanding of various substances, how they interact with the brain and body, which medicines are appropriate to specific behavioral health issues and conditions, and how to describe common physical experiences and risk factors. In the dosing sessions, they need to be aware of how to respond to symptoms that might represent adverse events including referrals to other disciplines as appropriate. Training in guidance and integration is also important. With changes in federal and state regulations related to clinical use of psychedelics, specific educational programs and certifications will be required for PAT practitioners, and chaplains should adhere to these standards. Developing these skills is best done under supervision, where live practice and feedback experience can be gained.

In psychedelic research and practice communities, there is controversy about whether PAT providers should be allowed to offer psychedelic care without having had their own psychedelic experiences. The argument is that one cannot competently prepare a participant for their non-ordinary state of consciousness without having one’s own sense of the mental, emotional, and spiritual landscape (Nielson & Guss, 2018). Again, given the current legal frameworks, this presents a problem for prospective care providers. While we agree that there are significant advantages to having personal experiences with non-ordinary states, it should not be mandated. Chaplains routinely provide support for people processing mystical or spiritual experiences, even when they have different spiritual or religious frameworks than the participants they support. This is a deeply personal choice, and chaplains must follow their own convictions, be aware of implications of psychedelic use within their faith-group endorsement (required for board certification), and manage their own health (Beachy & Petersen, 2022).

### Advocacy for Chaplains

At this point in the history of psychedelic care many researchers and practitioners are familiar with the mystical and spiritual encounters of participants, and they are aware of indigenous religious practices and communities (Celidwen et al., 2023; Urrutia et al., 2023). Unfortunately, they are frequently less comfortable with what to do about these realities (Mosurinjohn et al., 2023). Further, many in the psychedelic research communities are largely unaware of the presence of chaplains. This is common within the larger healthcare landscape as well (Balboni et al., 2022), and chaplaincy organizations have long called for advocacy for the role of chaplains to best serve the spiritual needs of patients as stakeholders themselves (Professional Advocacy, 2025).

As an increasing number of chaplains engage in PAT, there will be a need for them to find places of practice, which will require advocacy. For example, in June 2023 the FDA issued draft guidance for psychedelic research and treatment and opened a public comment period. This document defined the interdisciplinary caregivers who should be present for support in the various stages of psychedelic care, but did not include chaplains, which may neglect the SERT needs of patients (Center for Drug Evaluation & Research, 2023). In response to this, several organizations, including the Association of Professional Chaplains and Transforming Chaplaincy submitted response letters describing the clinical, ethical, and practical value of having chaplains involved in this space. Federal, state, and local regulatory agencies may overlook the inclusion of chaplains as certifiable facilitators without these kinds of efforts to highlight their role in PAT.

There are many psychedelic care certificate programs, but most do not include issues specifically related to spiritual care (Palitsky et al., 2025a). Given the abundance of evidence related to the mystical and spiritual experiences of participants in psychedelic care, we argue that this is a potentially dangerous oversight and one that neglects pathways of further healing and growth. SERT dynamics should be included in curricula by education programs, preferably led by chaplains as subject matter experts.

When research protocols are designed, the therapeutic support offered usually does not involve chaplains (Beachy & Petersen, 2022). Spiritual assessments prior to the dosing sessions are usually not part of the protocols. As in other areas of healthcare, chaplains working at research institutions will need to be proactive in identifying themselves to principal investigators and other team members. Being well versed in the evidence base and able to creatively argue for the benefit of spiritual care to participants will provide opportunities to develop more open protocols.

## Limitations

The authors recognize that while the rationale for chaplain involvement in PAT is sound and many of the existing professional chaplaincy competencies are readily transferrable, some significant limitations remain. Due to the heterogeneity of current psychedelic research protocols (Baker et al., 2023; Chisamore et al., 2024) and therapeutic support models (Brennan et al., 2023), there are no firm standards by which to evaluate the efficacy of chaplain support in the context of PAT. Further, because PAT is a nascent field, the competencies presented here are proposed based on current evidence, but lack full validation in clinical environments. More research is needed to evaluate facilitator interventions, spiritual screening models, and training methods. As more chaplains engage in PAT, the competencies discussed here will be tested and perhaps revised. Finally, because most psychedelics are not legally accessible, it is difficult for chaplains interested in engaging PAT to gain the clinical experience necessary to develop their skills.

## Next Steps

The future of PAT will likely continue to be characterized by rapid and disruptive change. As the FDA considers licensing more psychedelic substances, and as an increasing number of states consider legislation that will legalize or decriminalize the use of these medicines in clinical environments, chaplains seeking to provide care in these modalities will need to maintain vigilance to comply with legal and ethical frameworks. The authors encourage chaplains to pursue opportunities for learning via literature reviews, participation in communities of practice in the psychedelics community, and education that will prepare them well for practice when greater opportunities for practice present themselves. To date, no national or international standards of practice have been established. The authors encourage these SERT competencies to be included in standards established by legal and professional bodies that undertake these efforts. We further urge psychedelic facilitation education programs to incorporate these dynamics in their curricula to better equip future facilitators (Palitsky et al., 2025b). We also believe that the psychedelic research and clinical practice communities would do well to follow the interprofessional generalist/specialist model of care implemented in Palliative Care, which is inherently patient/participant-centered, holistic, and oriented toward addressing quality of life (Miller et al., 2023). Chaplaincy education and certification bodies will continue to hold the standards of specialist training and practice, and chaplains will make themselves available to collaborate with and educate our colleagues in SERT support.

Beachy and Petersen (2022) summarize the unique gifts that chaplains offer to this emergent field: “The capacity to embody a non-anxious presence in the midst of crisis is a cultivated skill possessed by seasoned chaplains and can support spiritual care practitioners who serve as guides for powerful psychedelic-induced experiences” (pp. 95–96). We eagerly anticipate the increasing opportunities to contribute to the healing and health of a population in need of support.
